# Ribosomal proteins, miRNAs, and extracellular vesicles in myelodysplastic syndromes: molecular regulation and biomarker potential

**DOI:** 10.3389/fonc.2026.1863527

**Published:** 2026-08-13

**Authors:** Gazmend Temaj, Sarmistha Saha, Brigitta Buttari, Pelin Telkoparan-Akillilar, Silvia Chichiarelli, Nexhibe Nuhii, Rifat Hadziselimovic, Luciano Saso

**Affiliations:** 1 Faculty of Pharmacy, College UBT, Prishtina, Kosovo; 2 Department of Biotechnology, GLA University, Mathura, Uttar Pradesh, India; 3 Department of Cardiovascular and Endocrine-Metabolic Diseases and Aging, Istituto Superiore di Sanità, Rome, Italy; 4 Department of Medical Biology, Faculty of Medicine, Gazi University, Ankara, Türkiye; 5 Department of Biochemical Sciences “A. Rossi-Fanelli”, Sapienza University of Rome, Rome, Italy; 6 Department of Pharmacy, Faculty of Medical Sciences, State University of Tetovo, Tetovo, North Macedonia; 7 Faculty of Science, University of Sarajevo, Sarajevo, Bosnia and Herzegovina; 8 Department of Physiology and Pharmacology “Vittorio Erspamer”, Sapienza University of Rome, Rome, Italy

**Keywords:** extracellular vesicle, lncRNA, MDS, microRNA, ribosomal proteins

## Abstract

Myelodysplastic syndromes (MDS) are a heterogeneous group of disorders caused by abnormalities at the hematopoietic stem cell level and are associated with ineffective hematopoiesis, peripheral cytopenias, and a propensity for leukemic transformation. Recent research elucidates the key mechanisms and roles of ribosomal proteins (RPs), microRNAs (miRNAs), extracellular vesicles (EVs), and long non-coding RNAs (lncRNAs) in the pathophysiology of MDS. Mutations or altered expression of RP genes lead to ribosomal stress and activation of p53, resulting in impaired erythropoiesis and apoptosis of hematopoietic progenitors. Dysregulation of miRNAs modulates gene expression programs that are essential for hematopoietic differentiation and apoptosis. Moreover, EV-associated miRNAs mediate multiple processes, including intercellular communication and regulation of the bone marrow (BM) microenvironment, thereby contributing to DNA damage and clonal evolution. EV-associated miR-10a and miR-15a have been reported to induce DNA damage in HSCs. LncRNAs have emerged as promising tools for cancer diagnosis and prognostic biomarkers. They play critical roles in regulating malignant cell proliferation, apoptosis, and epigenetic modifications. This overview highlights the molecular complexity of MDS pathogenesis, offering insights to support stratified diagnosis and the development of targeted therapies.

## 1 Introduction

**Figure 1:**
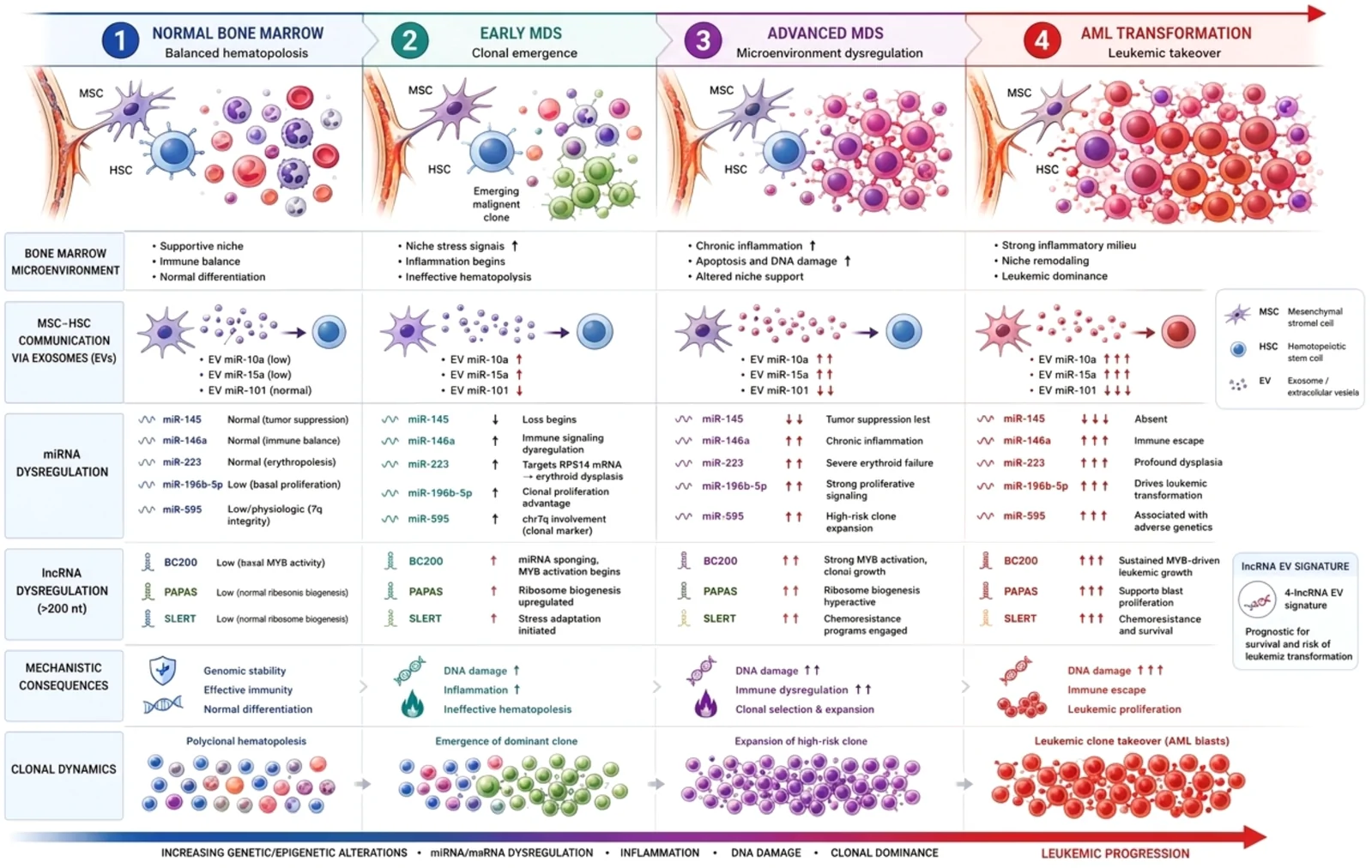
Clonal evolution from MDS to AML driven by non-coding RNA–mediated microenvironmental interactions. The figure depicts stepwise progression from normal bone marrow to AML, with increasing niche dysfunction, inflammation, and clonal dominance. MSC-derived extracellular vesicles transfer regulatory RNAs (↑miR-10a/miR-15a, ↓miR-101) that induce DNA damage and reprogram HSCs. Key miRNA alterations include miR-145 loss (impaired differentiation), miR-146a dysregulation (immune activation), miR-223 (erythroid defects), miR-196b-5p (proliferation), and miR-595 (7q-associated risk). lncRNAs BC200, PAPAS, and SLERT promote MYB activation, ribosome biogenesis, and chemoresistance. These changes drive genomic instability, ineffective hematopoiesis, expansion of malignant clones, and leukemic transformation.

Myelodysplastic syndromes (MDS) are a heterogeneous group of clonal hematopoietic disorders characterized by ineffective hematopoiesis, peripheral cytopenias, and an increased risk of progression to acute myeloid leukemia (AML) (1). The molecular pathophysiology of MDS is highly complex, involving an interplay between genetic mutations, dysregulated gene expression, and microenvironmental cues (2). The growing number of identified molecular abnormalities in MDS has the potential to significantly improve disease diagnosis, prognostic assessment, prediction of treatment responses, and how new targeted therapies are developed for affected patients (3) (**Figure 1**). In recent years, ribosomal proteins (RPs), microRNAs (miRNAs), extracellular vesicles (EVs), and long non-coding RNAs (lncRNAs) have emerged as key contributors to this complexity, offering novel insights into disease mechanisms and potential biomarkers for diagnosis and prognosis (2, 3).

Mutations in RP genes, such as RPS14, RPL5, and RPL11, induce ribosomal stress and activate p53, leading to cell cycle arrest and apoptosis in hematopoietic progenitors. Haploinsufficiency of specific RPs, as observed in the 5q-syndrome, disrupts ribosome assembly and impairs erythropoiesis (4), while overexpression of certain large subunit proteins, including RPL23, correlates with high-risk MDS phenotypes and poor clinical outcomes (5). These findings highlight the critical role of RP dysregulation in both disease initiation and progression. miRNAs further integrate ribosomal stress signals with hematopoietic regulation. Altered miRNA expression profiles in MDS patients influence p53 signaling and stress responses, modulate erythroid differentiation, and contribute to the abnormal proliferation of hematopoietic stem and progenitor cells (1, 6). For example, reduced levels of tumor suppressor miRNAs such as miR-145 and miR-146a (7), as well as overexpression of miR-223 (8), have been associated with erythroid dysplasia through targeting RP mRNAs. Extracellular vesicles serve as vectors for miRNAs and other cargo molecules, mediating intercellular communication within the BM microenvironment. EV-associated miRNAs, including miR-10a and miR-15a, can induce DNA damage in hematopoietic stem cells and promote mutagenesis, offering both mechanistic insight and potential non-invasive biomarkers for disease monitoring (9).

Long non-coding RNAs (lncRNAs) also play crucial roles in MDS pathogenesis. By modulating transcription, sponging miRNAs, and influencing epigenetic remodeling, lncRNAs affect hematopoietic differentiation, apoptosis, and therapeutic resistance. Specific lncRNA signatures carried by EVs have been suggested to be associated with patient survival and risk of leukemic transformation, independent of conventional clinical indices. In this review, we examine how RP mutations, miRNA dysregulation, EV-mediated signaling, and lncRNA dynamics collectively shape the molecular heterogeneity of MDS, and we discuss their emerging potential as biomarkers for patient stratification, prognostic assessment, and the development of targeted therapies (10).

## 2 Molecular mechanisms of miRNA biogenesis, function, and ribosome regulation in health and cancer

The discovery of the lin-4/lin-14 regulatory paradigm in *C. elegans* marked the first identification of microRNAs (11–15). MicroRNAs of viral origin (v-miRNAs) can act as post-transcriptional regulators of gene expression in host cells. The expression of viral microRNAs can prolong the lifespan of the infected cells. In prokaryotic cells, microRNAs are not naturally present and remain poorly understood in prokaryote virus interactions. Viral microRNAs are comparable in size to eukaryotic microRNAs and can be exported outside the cell via vesicles. Many of these v-miRNAs are involved in the biogenesis and regulation of target mRNAs (16). The biogenesis of the microRNAs occurs through two main pathways: canonical and non-canonical miRNA biogenesis (17). The biogenesis of miRNA begins with transcription of a primary miRNA (pri-miRNA) by Pol II (RNA polymerase), generating a transcript containing a characteristics stem-loop structure. The processing of pri-miRNAs occurs in the nucleus by a complex called the microprocessor (RNase III enzyme Drosha and cofactor DiGeorge Syndrome critical region 8 (DGCR8)). The pri-miRNA is cleaved by Drosha at the base of the hairpin, producing the pre-miRNA (precursor miRNA), with a main characteristic of a 2-nucleotide 3’ overhang. With the help of Exportin 5 (XPO5), the pre-miRNA is exported to the cytoplasm from the nucleolus in a Ran-GTP-dependent manner. After export to the cytoplasm, other enzymes such as RNase III and Dicer further process the pre-miRNA by removing the terminal loop. This process results in a miRNA duplex, one strand of which is preferentially loaded onto an Ago (Argonaute) protein to form the RNA-induced silencing complex (RISC), while the passenger strand is typically degraded (18, 19).

Maturation of the complex RISC includes the complex, which consists of Ago2, Staphylococcal nuclease domain-containing protein 1 (SND1), Astrocyte elevated gene-1 (AEG1), Fragile X mental retardation protein 1 (FMR1), Vasa intronic gene (VIG), RNA helicases Rm62 (R2D2 in Drosophila), and Armitage-RNA. All components of this multiprotein complex contribute to the recognition and repression of target mRNAs through translation inhibition or mRNA degradation (20, 21). Inside the nucleus, it is shown that miRNAs have been localized in visible subnuclear domains, such as the nucleolus and chromatin. Here, miRNAs participate in transcriptional gene activity (TGA) and transcriptional gene silencing (TGS) by modulating chromatin remodeling and epigenetic regulation at specific gene loci. These functions demonstrate that miRNAs regulate gene expression beyond cytoplasmic post-transcriptional control, extending their influence to a wide range of cellular processes (22, 23).

The tumor suppressor p53 plays a key role in miRNA processing by interacting with components of the microprocessor complex, including Drosha and the RNA helicases DDX5 and DDX17, thereby enhancing the maturation of specific miRNAs involved in cell cycle control and apoptosis. p53-mediated modulation of miRNA pathways also influences epithelial–mesenchymal transition (EMT), contributing to cancer progression and metastasis through miRNA-dependent mechanisms (24, 25).

All living organisms rely on ribosomes for protein synthesis, a fundamental and autonomous process that defines a key characteristic of life. During evolution, as cells increased in size and complexity, the number of ribosomes per cell also expanded. Ribosomes, cytoplasmic organelles present in every cell, are responsible for decoding RNA messages and translating them into the amino acid sequence to produce functional proteins. mRNA translation occurs in three stages: initiation, elongation, and termination. A key feature of the ribosome is its high fidelity in reading codons, reflected by the low frequency of missense, nonsense, and frameshift errors, which ensures accurate protein synthesis and proper protein function. The high precision of codon reading is ensured by the accurate base-pairing between codons and anticodons at the ribosomal A site (26, 27). Peptide bond formation occurs with high fidelity through the correct selection of aminoacyl-tRNAs. After translation, a proofreading mechanism further edits incorrectly formed peptidyl-tRNAs to maintain translational accuracy during termination. Peptide bond formation exhibits high fidelity through the accurate selection of aminoacyl-tRNAs. In addition, proofreading mechanisms operating during translation further enhance translational fidelity by eliminating incorrectly selected peptidyl-tRNAs during termination (28).

Ribosome biogenesis is closely linked to cell proliferation, a relationship that becomes dysregulated in cancer cells (29). This connection was first observed over 125 years ago by Pianese, who reported enlarged and distinct nucleoli in cancerous cells (30), highlighting alterations in ribosomal production associated with malignant growth. Several studies have investigated how ribosome biogenesis is altered in cancer cells, including disruptions at multiple levels such as transcription, ribosome processing and assembly, and translation (31, 32).

The ribosome is a fundamental component of the cellular machinery, playing a central role in protein synthesis. The identification of mutations in the RPs gene was unexpected, yet it has greatly advanced our understanding of numerous genetic disorders (33). For example, mutations in RPS19are associated with Diamond-Blackfan anemia (DBA), a congenital erythroid aplasia (34). This and other discoveries led to the concept of ribosomopathies, a group of diseases caused by defects in ribosomal proteins or ribosome biogenesis factors. Multiple studies have reported a strong association between ribosomal protein haploinsufficiency and human diseases (35).

Ribosomopathies encompass disorders caused by mutations or haploinsufficiency of RPs, leading to impaired ribosome assembly or function. Examples include DBA, resulting from mutations in several ribosomal proteins (26, 36); Treacher Collins Syndrome (TCS), caused by defects in rRNA transcription (37, 38); Shwachman-Diamond Syndrome (SDS), due to mutations in the SDS gene required for 60S ribosomal subunit biogenesis and maturation (39); the 5q-syndrome, associated with mutations in RPS14 haploinsufficiency resulting from del (5q) (4, 40); junctional epidermolysis bullosa (JEB), associated with a premature termination codon in the LAMB3 gene (41), and congenital dyskeratosis, caused by mutations in DKC1, a gene involved in rRNA modification (42, 43). Collectively, these diseases underscore the critical role of ribosomal integrity in maintaining cellular homeostasis and human health.

### 2.1 microRNAs: ribosome biogenesis, translation, and biomarkers

miRNAs play a pivotal role as regulators and potential biomarkers of ribosome biogenesis (RiBi), primarily through their interaction with RPs. miRNAs can target RP mRNAs by binding to the 3′ UTRs or coding regions, thereby modulating RP expression levels. This regulation influences ribosome assembly and can contribute to the production of specialized ribosomes with altered translational capacity (44). The regulatory effects may persist over hours to days, partly due to the delayed Argonaute loading, which also affects RiBi (3, 45). Thus, miRNAs exert a dual role, both upregulating and downregulating RPs, in a context-dependent manner, such as during different stages of the cell cycle, enabling adaptive control over RiBi processes (45).

miRNAs are closely associated with dysregulation of RiBi in various diseases, including cancer and ribosomopathies. Dysregulated RP-targeting miRNAs have been linked to defective ribosome biogenesis; for example, miR-145 downregulation is associated with osteosarcoma (44). Moreover, circulating miRNA profiles in body fluid can serve as non-invasive diagnostic tools, reflecting RiBi abnormalities, such as miR-320a, which has been proposed as a biomarker of defects in ribosome biogenesis (45). Changes in miRNA expression have significant prognostic value for clinical outcomes in both rare and cancerous diseases, such as gliomas, with reported sensitivities exceeding 80% (44, 45). In general, miRNAs serve as a critical link between molecular regulation of RPs and their clinical application, providing insights into translation-related pathologies while offering actionable biomarkers for diagnosis and prognosis (**Table 1**).

**Table 1:** MicroRNAs in ribosome biogenesis and disease: regulatory mechanisms, biomarkers, and clinical applications.

Category	Mechanistic role	Example miRNAs/diseases	Clinical relevance
Regulation of ribosome biogenesis	Direct targeting of RP mRNAs (3′ UTR or coding region) to fine-tune RP abundance and ribosome assembly, generating specialized ribosomes.	miR-145, miR-146a, miR-223; ribosomopathies and cancers	Links RP dosage to translational programs controlling proliferation and differentiation.
Temporal control of RiBi	Modulation of RiBi over hours to days due to delayed Argonaute loading and slow decay of RP proteins and assembly factors.	Context-dependent miRNAs regulating long-lived RPs/biogenesis factors	Shapes cellular adaptation to sustained stress and therapeutic exposure.
Dual regulatory roles	Context-dependent up- or down-regulation of RP expression and ribosome output according to cell-cycle phase and stress signals.	miRNA can act as tumor suppressor or oncogene in different settings	Explains apparently opposite roles of individual miRNAs in different disease contexts.
Disease associations	Dysregulated RP-targeting miRNAs contribute to defective RiBi and translational control in cancer and ribosomopathies.	miR-145 downregulation in osteosarcoma; miRNAs altered in ribosomopathies and MDS	Identifies candidate nodes for therapeutic modulation of ribosome biogenesis.
Biomarker (diagnostic) applications	Circulating miRNA profiles reflect RiBi status and translational stress in body fluids.	miR-320a indicating defects in ribosome biogenesis	Non-invasive tools for early detection of translation-related disorders.
Biomarker (prognostic) applications	Altered miRNA expression patterns correlate with clinical outcome and disease aggressiveness.	miRNA signatures in gliomas with high sensitivity (>80%)	Support risk stratification, prediction of progression, and response monitoring.
miRNA Regulation	miRNAs balance RiBi via oncogene targeting	miR-34a	suppresses *c-MYC*
Therapeutic Resistance	promotes 5-FU resistance	miR-21	upregulating the RiBi genes.
Metastasis	EMT leads to RiBi activation and association	miR-200	downregulation
Biomarker Potential	RiBi	miR-155	predict RiBi hyperactivity and nucleolar stress responses in tumors.

Non-Hodgkin’s lymphomas (NHLs) are a heterogeneous group of malignant lymphomas that can involve both lymph nodes and extranodal sites, with the BM being the most common site of extranodal involvement. Veryaskina et al. reported that several miRNAs, including miRNA-124, miRNA-221, and miRNA-15a, are significantly downregulated in NHL, whereas let-7a is significantly upregulated, showing more than 2-fold increase in BM compared to Anaplastic Large Cell Lymphoma (AL) and Non-Cancerous Blood Disease (NCBD). Analysis of these expression patterns revealed that the let-7a/miR-124 ratio exhibits high sensitivity, making these miRNAs promising biomarkers for distinguishing NHL from AL and NCBD. These findings suggest that miRNA expression profiling may serve as a valuable diagnostic tool in the early detection of NHL (46).

### 2.2 Ribosomal proteins and miRNAs in MDS pathogenesis

Mutations in RP genes make a major contribution to the pathogenesis of MDS, which is considered a ribosomopathy and is characterized by ineffective haematopoiesis and predisposition to leukemic transformation. Several research groups have reported mutations or the haploinsufficiency of the RP genes. Alterations in ribosomal proteins, such as RPS14, RPL5, and RPL11, reduce ribosome biogenesis and function, leading to ribosomal stress. Moreover, some RPs are involved in the activation of different signaling pathways, including the p53 pathway (6, 47, 48). Disruption of ribosome production affects protein synthesis fidelity and disrupts cellular homeostasis, ultimately contributing to the MDS phenotype. Similarly, to RP mutations, miRNAs also play a significant role in the pathogenesis and physiology of MDS. They exert their effects through the regulation of gene expression networks involving RPs, which are essential for haematopoietic differentiation, apoptosis, and cell proliferation. Dysregulation of miRNAs, such as miR-145 and miR-146a, affects key signaling pathways and interacts with different RPs, further contributing to BM failure and clonal evolution in MDS (49, 50). The combined dysregulation of RPs and miRNAs have a detrimental impact on the molecular landscape of MDS, highlighting their relevance for prognosis and therapeutic strategies.

Deletion of miR-595, located on chromosome 7q, occurs within a commonly deleted region associated with MDS characterized by monosomy 7(–7) or isolated 7q deletion (7q-). RPL27A has been identified as a target of miR-595. Downregulation of this RP leads to activation of p53, induction of apoptosis, and inhibition of cell proliferation. This p53-dependent effect also reduces the synthesis of the 60S ribosome subunits. RPL27A plays a pivotal role in nucleolar integrity as well as ribosome maturation and assembly. Increased expression of miR-595 has been associated with downregulation of target genes in MDS patient samples, together with RPL29A upregulation (51).

Other ribosomal proteins, such as RPS14, are expressed at reduced levels in patients diagnosed with MDS, as shown by immunohistochemical analysis of confirmed BM biopsies. Nie et al. demonstrated that overexpression of miR-223 in MDS patients is associated with cellular transformation and impaired erythroid differentiation, possibly through the regulation of RPS14 (52).

Wen et al. reported up-regulation of miR-196b-5p in intermediate II and high-risk groups, as well as in secondary acute myeloid leukemia (s-AML) patients, compared with controls, suggesting that increased expression is associated with a higher risk of MDS development. The same group observed alterations in cell proliferation and apoptosis following transfection of MDS cells with miR-196-5p mimics or inhibitors. Overexpression of miR196b-5p promoted cell proliferation, while apoptosis was reduced. Conversely, miR-196b-5p downregulation decreased proliferation and increased apoptosis. These findings suggest that miR-196b-5p overexpression contributes to an increased risk of leukemic transformation in patients with MDS (53) (**Table 2**).

**Table 2:** Molecular players in MDS: mechanisms, clinical correlates, and author notes.

Molecule	Mechanistic role in MDS	Clinical correlation	Author perspective/notes
RPS14	Haploinsufficiency in 5q-syndrome → ribosomal stress → p53 activation → apoptosis in hematopoietic progenitors; impaired erythropoiesis	Diagnostic for 5q-syndrome; prognostic for erythroid response	Keystone in MDS ribosomopathy; potential target for ribosome-restoring therapies
RPL5/RPL11	Haploinsufficiency → disrupted ribosome assembly → p53 activation → bone marrow failure and clonal evolution	Associated with bone marrow failure; possible predictor of leukemic progression	Key contributors to MDS initiation; need functional validation in MDS models
RPL23	Overexpression correlates with high-risk MDS and poor clinical outcomes	Marker of high-risk phenotype and poor prognosis	Suggests complex RP dysregulation; may reflect ribosomal subunit imbalance
RPL27A	Downregulated via miR-595 targeting → p53 activation → apoptosis and reduced large subunit synthesis	Relevant to −7/7q− MDS; linked to disease severity	miR-595/RPL27A axis is a critical node in 7q-deletion MDS
miR-145	Downregulation → impaired tumor suppression; affects p53 signaling and erythroid differentiation	Associated with increased proliferation and impaired differentiation	Loss may drive clonal evolution; candidate for miRNA replacement therapy
miR-146a	Downregulation → dysregulated innate immune signaling and stress responses	Linked to bone marrow failure and clonal expansion	Often co-downregulated with miR-145; potential dual-target biomarker
miR-223	Overexpression → targets RPS14 mRNA → exacerbated erythroid dysplasia	Associated with cellular transformation in MDS	Promising biomarker for erythroid dysfunction; therapeutic target
miR-196b-5p	Overexpression → promotes cell proliferation, reduces apoptosis; linked to leukemic transformation risk	Upregulated in intermediate-II/high-risk MDS and s-AML	Strong prognostic marker; potential driver of AML progression
miR-595	Located on chr7q; deletion/downregulation → RPL27A upregulation paradox; complex role in 7q− MDS	Relevant to monosomy 7 and 7q deletion MDS	Context-dependent effects; needs clarifying in MDS cohorts
EV-associated miR-10a/miR-15a	MSC-derived EVs transfer miRNAs → DNA damage, oxidative stress, mutagenesis in HSCs via p53 targeting	Correlate with disease stage and prognosis; non-invasive biomarkers	Key mediators of bone marrow microenvironment signaling; therapeutic targets
EV-associated miR-101	Reduced expression in high-risk MDS and AML/MDS with myelodysplasia-related changes	Biomarker of disease progression	Potential indicator of leukemic transformation risk
lncRNA BC200	Regulates malignant proliferation via miRNA sponging and MYB oncogene modulation	Associated with MDS cell growth	Novel therapeutic target; mechanistic studies needed
4-lncRNA EV signature	EV-carried lncRNAs associated with patient survival and leukemic transformation risk	Independent prognostic value beyond clinical indices	Robust signature for risk stratification; requires validation in large cohorts
rDNA-derived lncRNAs (PAPAS, SLERT)	PAPAS: represses rRNA synthesis under stress; SLERT: enhances pre-rRNA processing via DDX21 interaction	Dysregulation linked to nucleolar morphology and chemoresistance	Fundamental ribosome biogenesis regulators; potential in ribosome-targeting therapies

### 2.3 Well-characterized sncRNAs and lncRNAs in MDS: molecular pathways and clinical significance

Specific miRNAs, ribosomal protein defects, EV-associated non-coding RNAs, and lncRNAs contribute to distinct stages of MDS pathogenesis by disrupting HSC homeostasis, lineage commitment, and terminal differentiation. Importantly, many of these molecular alterations correlate with specific MDS subtypes and disease-risk categories defined by current prognostic systems, including the IPSS-R and IPSS-M.

Among low-risk MDS, particularly del(5q) disease, loss of miR-145 and miR-146a represents a characteristic molecular event. Their reduced expression leads to depression of TRAF6 and IRAK1, promoting inflammatory signaling, p53 activation, and ineffective erythropoiesis while impairing normal HSC differentiation. Consistent with these mechanisms, circulating miR-145 and miR-146a have demonstrated diagnostic value, with reported sensitivities exceeding 80% (7). Likewise, deficiency of ribosomal proteins, including RPS14 and RPL22, activates the nucleolar stress–p53 axis, a hallmark of ribosomopathy-associated MDS that contributes to HSC exhaustion, defective erythroid maturation, and clonal selection. Reduced RPL22 expression has been associated with impaired HSC differentiation and leukemic transformation in both human MDS and experimental models (60).

In contrast, progression toward higher-risk MDS and secondary AML is characterized by the accumulation of oncogenic non-coding RNA alterations. miR-223, which targets RPS14, exacerbates erythroid dysplasia while simultaneously influencing lineage commitment by promoting granulopoiesis at the expense of erythroid and monocytic differentiation through LMO2 repression (59). Similarly, miR-196b-5p is significantly upregulated in Intermediate-2 and High-risk MDS as well as secondary AML, where it enhances proliferation, suppresses apoptosis, and contributes to leukemic evolution (8, 54, 55). Loss of miR-595, frequently observed in MDS with monosomy 7 or isolated 7q deletion, further links cytogenetic abnormalities to ribosomal dysfunction through RPL27A downregulation and p53-dependent apoptosis (51).

The bone marrow microenvironment also undergoes progressive molecular remodeling during disease evolution. Mesenchymal stromal cell-derived EVs enriched in miR-10a and miR-15a induce DNA damage and mutagenesis in HSCs, and their abundance correlates with disease stage, supporting their potential as prognostic biomarkers (9). Conversely, reduced EV-associated miR-101 characterizes high-risk MDS and AML with myelodysplasia-related changes, reflecting disease progression (9). In parallel, EV-derived lncRNA signatures independently predict overall survival and leukemic transformation beyond conventional clinical parameters.

Beyond their biomarker value, lncRNAs actively regulate hematopoietic differentiation. BC200 promotes malignant cell proliferation through miRNA sponging and modulation of the oncogenic transcription factor MYB (56, 57). More broadly, lncRNAs regulate early hematopoietic commitment and multilineage differentiation, and disruption of these regulatory networks contributes to aberrant erythropoiesis, granulopoiesis, and megakaryopoiesis across MDS subtypes (58, 61). For example, the lncRNA Spehd is essential for multilineage differentiation, and its loss impairs progenitor cell metabolism and normal hematopoietic maturation (62).

Collectively, these observations demonstrate that non-coding RNA dysregulation is not merely a molecular hallmark of MDS but is closely associated with disease subtype, cytogenetic background, and molecular risk stratification. Low-risk MDS is predominantly characterized by alterations affecting ribosomal function and ineffective erythropoiesis, whereas higher-risk disease progressively acquires non-coding RNA and EV-mediated signaling networks that promote clonal expansion, remodeling of the BM niche, and leukemic transformation. These integrated mechanisms support the development of non-coding RNA-based biomarkers and targeted therapeutic strategies tailored to different stages of MDS progression.

## 3 Recent insights in lncRNAs and RiBi

Long non-coding RNAs (lncRNAs) play a pivotal role in the regulation of ribosome biogenesis, contributing to these complex processes and controlling cell growth and protein synthesis. Over the past decade, advances in transcriptomics and functional genomics have shown that lncRNAs interact with both the molecular machinery and the regulatory networks governing ribosome production, processing, and assembly (63, 64). Several lncRNAs are transcribed from ribosomal DNA (rDNA) loci or from regions proximal to rDNA clusters. These rDNA-derived lncRNAs can modulate the activity of RNA polymerase I (Pol I), the enzyme responsible for rRNA synthesis. For instance, the lncRNA PAPAS (Promoter and Pre-rRNA Antisense) is upregulated under nucleolar stress and recruit chromatin remodeling complexes to rDNA promoters, thereby leading to transcriptional repression of rRNA genes. This mechanism allows cells to swiftly downregulate rRNA synthesis in response to stress or metabolic cues (65, 66).

lncRNAs are also involved in post-transcriptional processing of pre-rRNA. For example, the lncRNA SLERT (SnoRNA-ended lncRNA enhancing rRNA transcription) interacts with the DEAD-box RNA helicase DDX21, thereby promoting efficient pre-rRNA processing and enabling ribosome assembly and maturation. Disruption of SLERT impairs ribosomal maturation, highlighting its essential role in maintaining the fidelity of ribosome biogenesis (65).

Disruption of lncRNAs that play pivotal roles in ribosome biogenesis has been increasingly linked to a variety of human diseases, especially cancer and ribosomopathies. Aberrant expression of lncRNAs such as SLERT and PAPAS has been associated with alterations in nucleolar morphology, dysregulated cell proliferation, and resistance to chemotherapeutic agents targeting ribosome biogenesis (66). In several cancer types, lncRNA-driven enhancement of ribosome production can lead to nucleolar hypertrophy and increased protein synthesis, thereby supporting oncogenic growth. Conversely, loss of function of the lncRNAs that normally repress rRNA transcription may contribute to ribosomopathies by impairing ribosome production and cellular homeostasis (10).

Due to their nucleolar localization and high specificity, lncRNAs represent promising biomarkers for diseases characterized by dysregulated ribosome biogenesis. Their cell- and tissue-specific expression patterns make them attractive candidates for the development of diagnostic tools and targeted therapies. Moreover, functional modulation of lncRNAs using antisense oligonucleotides or small molecules may provide novel strategies to control ribosome biogenesis in cancer or to correct ribosomal defects. Several studies have highlighted the role of lncRNAs as biomarkers in MDS, demonstrating their significant potential in diagnosis, prognosis, and therapeutic targeting. Distinct lncRNA expression profiles have been associated with disease progression and patient outcomes. For example, a 4-lncRNA signature has shown prognostic value independent of classical clinical parameters, with higher expression levels correlating with poor outcomes and increased rates of leukemic transformation (67, 68).

Functional studies further underscore the role of lncRNAs such as BC200, which regulate malignant cell proliferation through mechanisms including miRNA sponging and modulation of oncogenes such as MYB, a key driver of MDS cell growth (56). In addition, integrative analyses have demonstrated that lncRNAs influence critical processes such as hematopoietic differentiation, apoptosis, and epigenetic remodeling, all of which are central to MDS pathogenesis (67). Overall, these findings support the use of lncRNAs as valuable tools for risk stratification in patients diagnosed with MDS and as potential guides for the development of personalized therapeutic strategies.

## 4 Ribosomal RNA in MDS: roles of variants and modifications in human disease

Ribosomal RNA (rRNA) heterogeneity refers to the presence of sequence variations and chemical modifications within rRNA molecules, as well as differences in ribosomal protein composition, which together generate distinct ribosome subtypes in human cells. Recent studies have demonstrated that this heterogeneity varies across tissues and developmental stages and is dynamically regulated under physiological and pathological conditions. Tissue-specific rRNA sequence variants can contribute to the generation of specialized ribosomes with selective mRNA translation capacity, thereby influencing gene expression programs relevant to disease processes. In addition to sequence variability, post-transcriptional modifications of rRNAs, which are widely present in eukaryotes, further contribute to ribosome diversity and significantly impact translational efficiency and fidelity. Notably, these variations and modifications are often located at critical functional sites of the ribosome, such as regions involved in mRNA decoding and peptide bond formation, suggesting a direct role in cellular pathophysiology. In disease contexts, particularly in cancer and ribosomopathies, alterations in rRNA variant expression and ribosome composition have been associated with gene dysregulation, abnormal cell growth, and tissue-specific pathological phenotypes. For example, specific rRNA variants and mutations in RP have been linked to disorders such as Diamond-Blackfan anemia and increased susceptibility to tumorigenesis. The dynamic regulation of different rRNA variants and modifications during development, as well as in response to environmental and cellular stress, further underscores the importance of rRNA heterogeneity in both health and disease. These findings highlight the potential of targeting specific ribosome subtypes as a novel therapeutic strategy (69, 70).

### 4.1 Ribosome-extracellular vesicle

Extracellular vesicles (EVs) are lipid bilayer-enclosed particles with diameters ranging from nanometers to micrometers. In recent years, they have attracted increasing attention as key mediators of cell-cell communication (71, 72). EVs are classified into several categories based on size and biogenesis, including exosomes (~30–150 nm), exomeres (<50 nm), migrasomes (500–3000 nm), exopheres (up to several micrometers), and apoptotic bodies (50–5000 nm) (73).

Exosomes are small EVs generated by internal budding of late endosomal membranes, resulting in the formation of intraluminal vesicles that are released into the extracellular space. They carry a diverse cargo, including mRNA, microRNA, proteins, and, in some cases, ribosomal RNA, which can influence gene expression and ribosomal activity in recipient cells. Exosomes interact with target cells through mechanisms such as membrane fusion, endocytosis, or micropinocytosis, thereby enabling the delivery of functional biomolecules. Through these processes, exosomes can transfer mRNA that are molecules and proteins that are either translated in recipient cells or modulate translational machinery, thereby altering protein synthesis and cellular phenotype. This intercellular communication contributes to various biological processes, including cancer progression, immune regulation, and tissue-specific responses. Recent studies have highlighted the role of exosome-mediated transport of ribosomal components in reprogramming recipient cells. By delivering mRNA for translation and regulatory RNAs that affect mRNA stability and translation efficiency, exosomes can induce significant changes in cellular behavior, underscoring their impact on ribosome-associated mechanisms and gene expression regulation (74, 75).

In this context, EVs are involved in a wide range of cellular processes, including physiological regulation, immune response, neurological functions, cardiovascular activities, and viral pathogenesis (71, 76). The classification of exosomes remains not fully standardized; however, their molecular composition is often broadly divided into two categories. The first includes common components (77), such as proteins involved in membrane trafficking and fusion (Ras-associated binding GTPase, annexin, and heat shock proteins), as well as multivesicular bodies(MWB)-related proteins. tetraspanins (four-transmembrane domain proteins) and cytoskeletal or adhesion proteins, including integrins, actin, and myosin (78). The second category includes more specific components, which can vary depending on the cell of origin and physiological or pathological conditions. Examples include cGMP-dependent kinase 1 and x-box-binding protein 1, both detected in plasma-derived exosomes (79).

Both EVs and miRNAs are involved in the pathophysiology of MDS and play pivotal roles in intercellular communication within the BM niche and modulating hematopoietic stem cell (HSC) function. In MDS, EVs released by mesenchymal stromal cells (MSCs) carry specific miRNA cargo that can induce DNA damage, oxidative stress, and mutagenesis in HSCs, contributing to ineffective haematopoiesis and leukemogenesis (80). Particularly, miRNAs such as miR-10a and miR-15a, enriched in MSC-derived EVs from patients with MDS, modulate signaling pathways involving TP53 and other regulators of the cell cycle and apoptosis, including the tumor suppressor protein P53 (TP53), thereby influencing HSC fate and disease progression (81, 82). Moreover, distinct EV-associated miRNA profiles have been correlated with disease stage and patient prognosis, supporting their potential use as diagnostic and prognostic biomarkers as well as therapeutic targets (83, 84). Overall, these findings suggest that EV-associated miRNAs are a crucial mediator in the pathophysiology of MDS via epigenetic and post-transcriptional regulation of gene expression, and they provide a framework for the development of miRNA-based therapeutic strategies aimed at restoring normal haematopoiesis.

EVs and miRNAs hold strong promise as diagnostic tools in a wide range of diseases, including MDS. Their ability to reflect disease status and progression through non-invasive sampling makes them particularly attractive in clinical settings. EVs derived from BM mesenchymal stromal cells (BM-MSCs) in patients with MDS carry distinct miRNA profiles that correlate with disease severity and clinical risk. For instance, reduced expression of EV-associated miR-101 has been observed in high-risk MDS and in AML/MDS with myelodysplasia-related changes, highlighting its potential as a biomarker of disease progression (85).

Circulating exosomal miRNAs, including those specifically enriched in patients with MDS, have also been shown to distinguish MDS from aplastic anemia, providing a more detailed molecular stratification of these hematologic disorders (9, 86). Furthermore, EV-associated miRNAs, such as miR-10a and miR-15a, may modulate hematopoietic stem and progenitor cell function, influencing disease dynamics. Importantly, the expression levels of these miRNAs in plasma or BM samples provide prognostic and predictive value, enabling patient risk stratification and supporting personalized therapeutic monitoring (87, 88). The remarkable stability of EV-associated miRNA in biological fluids, together with their accessibility, makes them highly promising candidates for clinical application in early diagnosis, prognosis, and evaluation of treatment response in MDS.

### 4.2 Integrated RP–miRNA–EV–lncRNA network driving clonal evolution and resistance in MDS

The functions of the molecular targets discussed in MDS --RPs, miRNAs, EVs, and lncRNAs- are deeply interconnected, forming a unified network that directly drives clonal HSC expansion, leukemic progression, and therapeutic resistance: RP haploinsufficiency (e.g., RPS14, RPL5, RPL11) triggers ribosomal stress and p53 activation, while miRNA dysregulation reinforces this axis through targeting RP mRNAs (miR-223→RPS14, miR-595→RPL27A) and modulating p53 signaling (miR-145/miR-146a downregulation), creating a feedback loop that selects for p53-evading clones; EVs propagate these signals across the BM niche by transporting miR-10a/miR-15a that induce DNA damage and TP53 suppression in HSCs, promoting mutagenesis and clonal advantage, while carrying lncRNA signatures (e.g., 4-lncRNA EV signature) that predict leukemic transformation; lncRNAs further integrate epigenetic remodeling, miRNA sponging (BC200 sponges miRNAs to upregulate MYB), and ribosome biogenesis control (PAPAS, SLERT), driving therapeutic resistance and aggressive disease. A high-resolution visual model should explicitly integrate three converging layers of MDS pathobiology: First, RP haploinsufficiency or mutation in hematopoietic stem and progenitor cells induces nucleolar stress and the canonical p53 pathway through the ribosome–MDM2 checkpoint, leading to defective erythropoiesis, impaired HSPC maintenance and stem/progenitor cell attrition (89). Second, p53-centered stress signaling within the BM niche is further amplified by inflammatory and stromal cell-derived cues, thereby exacerbating ineffective haematopoiesis and promoting disease progression in MDS. Third, EV-mediated miRNA trafficking enables bidirectional communication between dysplastic hematopoietic cells and BM stromal cells, facilitating the transfer of regulatory miRNAs that reprogram transcriptional networks and lineage commitment throughout the marrow microenvironment (9). Accordingly, the integrated schematic (**Figure 1**) illustrates the bidirectional communication between RP-defective clones, BM niche cells, and HSPCs through bidirectional signaling pathways (90). It highlights EV-associated miRNAs as critical modulators of p53-dependent apoptosis, proliferation, and differentiation (91), and depicts how the convergence of RP defects, p53 activation, EV-mediated signaling, and lncRNA dysregulation culminates in the characteristic pathological features of MDS, including ineffective erythropoiesis, impaired myelopoiesis, abnormal megakaryocytic differentiation, clonal evolution, and progression toward AML.

### 4.3 Piwi-RNA

PIWI-interacting RNAs (piRNAs) and their associated PIWI protein have emerged as potential regulators of ribosome biogenesis through multiple mechanisms that influence RNA stability and translational control. PIWI proteins, belonging to the Argonaute subfamily, form RNA-induced silencing complexes with piRNAs to regulate gene expression by guiding sequence-specific repression or cleavage of target RNAs (92).

Recent studies have shown that the PIWI protein can directly interact with components of the ribosomal machinery and polysomes, suggesting that piRNAs contribute to the regulation of mRNA translation and ribosome activity (93). For example, the mammalian PIWI protein MIWI has been found in complexes with piRNAs and mRNAs within ribonucleoprotein particles and polysomes, indicating a role in translational control during gametogenesis (94). This association may influence ribosome biogenesis by modulating the stability and translation of mRNAs encoding ribosomal proteins and factors essential for ribosomal assembly. In addition, piRNAs are involved in epigenetic regulation of nucleolar genes that control rRNA transcription, thereby affecting ribosome production at the transcriptional level. Studies in Drosophila and mammalian systems have demonstrated that piRNA pathways can induce heterochromatin formation and DNA methylation at specific loci, leading to repression or fine-tuning of repetitive elements and rDNA clusters that regulate nucleolar function and ribosome synthesis (95, 96). This epigenetic control is crucial for maintaining genome stability and proper nucleolar activity, both of which are fundamental for efficient ribosome biogenesis.

Overall, the piRNA pathway links small RNA-mediated gene silencing with ribosome biogenesis by regulating both the transcriptional landscape of rRNA genes and the translational efficiency of ribosome-associated mRNAs. This multifaceted role highlights the importance of piRNAs and PIWI proteins beyond transposon silencing, extending to the regulation of core eukaryotic protein synthesis machinery (97). Understanding piRNA functions in ribosome biogenesis could therefore provide new insights into germline development, stem cell biology, and diseases associated with ribosomal dysfunction.

## 5 Discussion

Myelodysplastic syndromes (MDS) are heterogeneous clonal hematopoietic disorders characterized by ineffective hematopoiesis, cytopenias, and a high risk of progression to AML. Recent work connects MDS pathogenesis to RP defects, dysregulated miRNA, and EV-associated small non-coding RNAs (sncRNAs) and lncRNAs, which also hold biomarker potential (98, 99) (**Figure 1**). Key findings show that RP gene haploinsufficiency, particularly RPS14 in the 5q-syndrome, triggers p53-mediated apoptosis in hematopoietic progenitors, while overexpression of components like RPL23 is linked to high-risk disease and poor-prognosis phenotype (4, 47, 100, 101).

Folgado-Marco et al. (98) showed that haploinsufficiency of RPs disrupts the balance between ribosome biogenesis and translation, thereby aggravating BM failure and predisposing to clonal evolution. In addition, overexpression of the large subunit RPL23 has been associated with high-risk MDS phenotypes and poor prognosis, highlighting the complexity of RP dysregulation in disease progression (101).

miRNAs, in a complex regulatory network, are also involved in the control of gene programs governing hematopoietic differentiation and apoptosis, exerting both direct and indirect effects on ribosome biogenesis and function. Micheva and Atanasova (83) identified altered miRNA expression profiles in patients with MDS that modulate the p53 signaling pathway and stress response pathways linked to ribosomal dysfunction. In particular, reduced levels of tumor suppressor miRNAs such as miR-145 and miR-146a are associated with increased proliferation and impaired differentiation of hematopoietic stem and progenitor cells (83, 102). miRNA dysregulation also plays a pivotal role in erythroid lineage commitment by regulating RP expression. For example, overexpression of miR-223 in MDS has been reported to suppress RPS14 expression, thereby exacerbating erythroid dysplasia (52). Overall, miRNAs integrate ribosomal stress signals with hematopoietic regulation, influencing disease phenotype and clinical outcomes.

During intercellular communications with the BM microenvironment, EVs serve as a carrier of miRNAs, shuttling small regulatory molecules between stromal and hematopoietic cells. Meunier et al. (9) showed that EVs derived from mesenchymal stromal cells of patients with MDS can transport miRNAs such as miR-10a and miR-15a, which may contribute to DNA damage and promote mutagenesis in hematopoietic stem cells. These EV-associated miRNAs correlate with disease stages and prognosis, highlighting their potential as non-invasive biomarkers for disease monitoring and progression (9, 86). The remarkable stability and accessibility of EV-miRNAs in biological fluids further enhance their clinical value for diagnostic and prognostic applications in MDS.

The lncRNAs are also implicated as important epigenetic regulators and biomarkers in MDS. Yao et al. (68) identified a 4-lncRNA EV signature associated with patient survival and risk of leukemic transformation, independent of conventional clinical parameters. Several studies have shown that lncRNAs such as BC200 regulate malignant cell proliferation through mechanisms including miRNA sponging and transcriptional control of oncogenes such as MYB. In addition, Georgoulis et al. (67) demonstrate that lncRNA dysregulation significantly affects hematopoietic differentiation, apoptosis, and epigenetic remodeling, emphasizing its contribution to MDS pathogenesis and therapeutic resistance.

From a clinical perspective, the integration of these molecular biomarkers improves patient stratification, enhances prognostic accuracy, and supports the identification of more effective therapeutic strategies. The combination of mutations in RP, dysregulation of RNA synthesis, altered EV cargo, and lncRNA dynamics highlights the molecular complexity underpinning MDS. Collectively, these alterations disrupt normal haematopoiesis by impairing ribosome biogenesis, deregulating gene expression, reducing translation fidelity, and modulating microenvironmental signaling. These interconnected mechanisms ultimately promote clonal expansion and malignant transformation.

## 6 Conclusion and future perspectives: translational potential in MDS

The molecular insights summarized here, particularly the roles of RPs, miRNAs, EVs, and lncRNAs, open multiple translational avenues for MDS. While mechanistic understanding has advanced rapidly, several key challenges must be addressed before these discoveries can be integrated into routine clinical practice. Molecular research has significantly advanced our understanding of MDS, highlighting the central roles of RPs, miRNAs, EVs, and lncRNAs in disease pathogenesis. These factors represent promising diagnostic and prognostic biomarkers, as well as potential therapeutic targets. However, substantial challenges remain in translating these findings into routine clinical practice. Future research should prioritize a deeper mechanistic understanding of molecular crosstalk. In particular, the interactions between ribosomal protein dysfunction, miRNA regulatory networks, EV-mediated intercellular communication, and lncRNA-driven epigenetic control require further investigation to clarify the hierarchical and temporal dynamics that drive MDS progression. Large-scale, prospective studies are also needed to validate the sensitivity, specificity, and reproducibility of miRNA, lncRNA, and EV-based biomarkers, with the ultimate goal of developing standardized assays suitable for clinical application. Building on these molecular insights, novel therapeutic approaches aimed at restoring ribosomal function, normalizing miRNA expression, and modulating EV cargo should be developed and evaluated in preclinical models and clinical trials, with careful assessment of efficacy, delivery strategies, safety, and resistance mechanisms. Finally, the integration of multi-omics approaches and single-cell transcriptomic and proteomic profiling will enable a more precise characterization of MDS heterogeneity, including the identification of rare subclones, thereby supporting the development of personalized medicine strategies. Collectively, these advances have the potential to transform MDS management by enabling early diagnosis, improved risk stratification, tailored therapies, and better patient outcomes.
